# One-Step Electrodeposition of Hybrid Semiconductive CdSe/Nitrogen-Doped Carbon Dots Thin Films

**DOI:** 10.3390/ma18245691

**Published:** 2025-12-18

**Authors:** Katerina Pappa, Maria Myrto Dardavila, Athanasios Tzanis, Adamantia Zourou, Christina Mitzithra, Stylianos Hamilakis, Zaphirios Loizos, Konstantinos Kordatos, Constantina Kollia

**Affiliations:** 1 Laboratory of General Chemistry, School of Chemical Engineering, National Technical University of Athens, 9, Iroon Polytechniou Str., Zografou Campus, 15780 Athens, Greece; rinapap6@gmail.com (K.P.); ttzanis@hotmail.com (A.T.); mitzithra.christina@gmail.com (C.M.); zaphirisloizos@gmail.com (Z.L.); dinak@chemeng.ntua.gr (C.K.); 2 Laboratory of Inorganic and Analytical Chemistry, School of Chemical Engineering, National Technical University of Athens, 9, Iroon Polytechniou Str., Zografou Campus, 15780 Athens, Greece; adamantia_zourou@outlook.com.gr (A.Z.); kordatos@mail.ntua.gr (K.K.); 3 Laboratory of Organic Chemistry, School of Chemical Engineering, National Technical University of Athens, 9, Iroon Polytechniou Str., Zografou Campus, 15780 Athens, Greece; hamil@chemeng.ntua.gr

**Keywords:** hybrid semiconductive thin films, cadmium selenide, nitrogen-doped carbon dots, one-step electrocodeposition, photoelectrochemical cell, pulse current electrodeposition

## Abstract

Novel hybrid semiconducting thin films comprising CdSe with the addition of nitrogen-doped carbon dots (NCDs) were developed onto titanium substrates using a one-step electrocodeposition technique. The deposition took place using an acidic aqueous electrolytic bath containing hydrothermally produced ΝCDs under direct and pulse current regime. The specimens were studied using XRD, SEM-EDS, and UV-Vis spectroscopy techniques to determine their microstructural characteristics, surface morphology and composition and the energy gap, respectively. Their photochemical behavior was studied utilizing a photoelectrochemical cell (PEC). Variations in physical properties, along with significantly improved photoelectrochemical responses, were observed for the NCD-infused semiconductive thin films compared to their plain CdSe counterparts. These variations were highly affected by the incorporation rate of the NCDs in each thin film, as well as the imposed electrolysis conditions.

## 1. Introduction

Semiconducting chalcogenide materials continue to attract attention due to their tunable optoelectronic properties and their broad applicability in photovoltaics, photodetectors, and photocatalytic systems [1,2,3,4,5,6]. Beyond single-component semiconductors, hybrid systems that integrate inorganic chalcogenides with carbon-based nanostructures have emerged as a powerful means of enhancing photophysical behavior, modifying charge-transfer pathways, and improving device efficiency [7,8,9,10,11,12]. Such materials often display synergistic functionalities arising from interfacial interactions between the inorganic semiconductor and the carbon phase. For instance, the incorporation of carbon dots (CDs) or N-doped carbon dots (NCDs) into TiO_2_, ZnO, CdS, and NiFeOx has been shown to improve visible-light absorption, suppress electron–hole recombination, and promote more efficient photocatalytic or photovoltaic performance [13,14,15,16,17,18,19,20,21,22].

Cadmium selenide (CdSe) is a direct band-gap semiconductor (Eg ≈ 1.7 eV) with strong absorption in the visible region, making it a promising material for solar-energy harvesting, photoelectrochemical (PEC) systems, and optoelectronic devices [1,2,3,23]. When produced electrochemically, CdSe typically crystallizes in the metastable cubic zinc-blende phase with preferred (111) orientation [1,2,3], and its crystallographic texture and defect structure can be tailored by controlling the deposition potential or current waveform [1,24,25,26]. Carbon dots, and particularly nitrogen-doped CDs, offer an attractive route to enhance CdSe’s performance due to their environmentally friendly aqueous synthesis, strong visible-light photoluminescence, and favorable electronic properties associated with pyridinic and pyrrolic nitrogen centers [18,19,21,27,28].

Electrodeposition provides a particularly appealing route for synthesizing semiconductor thin films and hybrid structures because it is low-cost, aqueous-based, environmentally benign, and highly controllable [1,2,3,14,24,25,26]. Pulse-current (PC) electrodeposition offers additional control of nucleation and growth through alternating on/off cycles. Adjusting pulse frequency and duty cycle modifies ion diffusion, relaxation times, and surface adsorption phenomena, enabling grain refinement, modification of crystallographic orientation, and suppression of concentration gradients at the electrolyte–substrate interface [13,14,15,16].

In the present work, we combine electrochemically synthesized CdSe with environmentally benign N-doped carbon dots to develop hybrid CdSe/NCD thin films via a one-step electrocodeposition route under both direct-current (DC) and pulse-current regimes. A hydrothermally prepared NCD dispersion [29] was introduced into an acidic CdSe electrolyte previously established by our group [1,2,3]. The structural, morphological, compositional, optical, and photoelectrochemical properties of the resulting hybrid films were systematically investigated. To the best of our knowledge, this is the first report on the one-step electrochemical co-deposition of CdSe with N-doped carbon dots.

## 2. Materials and Methods

### 2.1. Production of the NCDs Solution

The solution containing NCDs, used to produce the thin films, was prepared by our scientific team. Detailed information about the fabrication process, as well as the characterization of the NCDs can be found in an article published by Falara et al. (2025) [29]. In short, NCDS were fabricated by using urea (≥99.5%, Sigma-Aldrich, Hamburg, Germany) and citric acid (≥99.5%, Sigma-Aldrich, Hamburg, Germany) via the hydrothermal method. Specifically, 3 g of urea and 3 g of citric acid are diluted in 10 mL distilled water by magnetic steering for approximately 10 min at room temperature. After transferring the solution into a stainless-steel reactor lined with Teflon, the latter is heated at 200 °C for twelve hours. The resulting solution is left to reach room temperature, then is subdued to centrifugation at 6000 rpm for 30 min and filtrated through a microporous membrane. This is necessary for the effective removal of large particles and other residues [18,30].

### 2.2. Production of the Hybrid Semiconductive Thin Films

The CdSe thin films were deposited potentiostatically under DC and PC conditions using a Metrohm’s Autolab PGSTAT302N MBA potentio-scan system (Metrohm AG, Herisau, Switzerland). More specifically, a conventional three-electrode setup was used, consisting of a Ti cylinder as the working electrode, a platinum-plated grid as the counter electrode and as reference a Silver/Silver Chloride Electrode—SCE (Ag/AgCl, Hanna Instruments, Cluj-Napoca, Romania). The titanium cylinder had a diameter of 12 mm and was rotating at 500 rpm during the electrodeposition. Also, the working electrodes were covered circumferentially with proper insulating material prior to electrodeposition. The applied potential during electrolysis, namely −0.6 V/SCE and −0.7 V/SCE, was determined using the cyclic voltammetry method. Under PC plating, three values of pulse frequency (*ν*) were examined, namely 0.1 Hz, 1 Hz, and 10 Hz, at each formerly mentioned voltage. The duty cycle was set to 70%, as suggested by our previous work [1].

All chemicals used for the preparation of the electrolytic bath were of analytical grade. The bath consisted of an aqueous solution containing 0.2 mol/L Cadmium sulfate (CdSO_4_, 99% purity, Chembiotin, Voula, Greece) and 2∙10^−3^ mol/L selenium dioxide (SeO_2_, 98% purity, Acros Organics, Waltham, MA, USA). The concentration of SeO_2_ was maintained at a low level to prevent the passivation of the titanium substrate, which can occur through the formation of a red insulating thin layer of selenium, while also ensuring the avoidance of excessive selenium deposition on the thin film [2,3,31].

Inspired by the work of Yang et al. [14] and from our own experience testing other concentrations, it was decided that 3.55 mL of NCDs solution should be added per 300 mL of electrolytic bath.

Electrolyte temperature was maintained constant at 85 ± 1 °C at all instances. Furthermore, the pH was adjusted to 2.2 by adding 1 M sulfuric acid solution (H_2_SO_4_, 1M, Fisher Chemical, Leicestershire, UK) into the electrolytic bath.

Also, plain CdSe films, i.e., without NCDs addition, were produced at each electrodeposition condition, which were used as reference samples.

The electrodeposition parameters are provided in Table 1. The total charge passed during each deposition was recorded coulometrically, and the current response was monitored throughout the process. The incorporation of NCDs induced a small decrease in the deposition current, a behavior commonly associated with carbon-based additives that alter interfacial electrochemical kinetics. This reduced current led to a slightly smaller amount of CdSe being deposited, which explains the decrease in thickness observed in the hybrid films. The resulting coatings exhibited thickness values of approximately 1 to 4 μm (Table 1), as estimated by the 2nd Faraday law, which states that the mass *m* of a substance deposited at an electrode is proportional to the total charge passed during electrolysis. The deposited mass is given by:

(1) $$m=\frac{Mr}{n}\cdot \frac{I\cdot t}{F}$$

where *Mr* is the molecular weight of the depositing system, *n* is the ionic charge, *I* is the applied current, *t* is the deposition time, and *F* is Faraday’s constant. The calculated mass was converted to film thickness using the density of electrodeposited cubic CdSe (5.74 g/cm^3^).

### 2.3. Characterization of the Hybrid Semiconductive Thin Films

The crystalline development of the thin films was determined using the X-ray diffraction (XRD) technique by employing a Bruker D8 Advance instrument (Bruker AXS GmbH, Karlsruhe, Germany) with a Cu*K*_α_ X-ray source. The recorded 2*θ* values spanned from 10° to 60°, and data was collected with a step size of 0.1° and a scanning time of 1 s per step.

Surface morphology details of the thin films were acquired through scanning electron microscopy (SEM) imaging utilizing a JEOL IT500LV instrument (JEOL Ltd., Tokyo, Japan). The electron beam was accelerated by applying 20 kV voltage. Elemental composition analysis of the specimens was performed utilizing an energy dispersive spectroscopy (EDS) detector, specifically the X-Max Extreme silicon drift detector manufactured by Oxford Instruments (Abingdon, UK).

For the determination of the energy gap of the thin films, a JASCO V-770 double-beam UV-Vis/NIR spectrophotometer (JASCO Corporation, Hachioji, Japan) was utilized. The spectrophotometer was equipped with an integration sphere, ISV-922/ISN-901i 60 mm (KGW-ISOTHERM, Karlsruhe, Germany), to capture the reflection spectrum. Measurements covered wavelengths ranging from 300 nm to 1400 nm. The scanning speed was set to 400 nm/min, resulting in a continuous spectrum.

**Figure 1:**
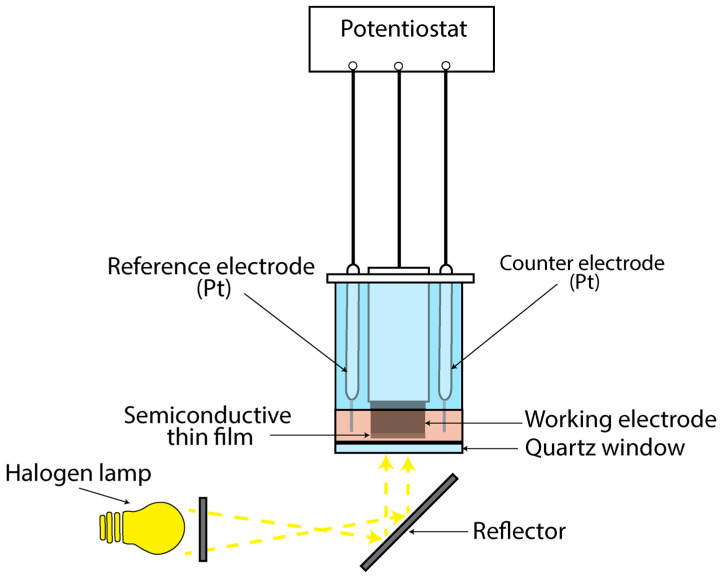
Schematic representation of the PEC used to study the thin semiconductive films’ photo-response.

To study the thin films’ photoresponse, a photo-electrolytic cell (PEC) was used (Figure 1). An alkaline sulfide–polysulfide electrolytic solution consisting of 1 M NaOH (Lach-Ner, 99.6%, Neratovice, Czechia), 1 M $Na_{2}S$ (Alfa Aesar, ≥60%, Ward Hill, MA, USA) and 1 M S (Fluka, ≥99.5%, Buchs, Switzerland) was employed as the working redox electrolyte. The experimental setup involved a three-electrode configuration, with two platinum rods serving as reference and counter electrodes, and the titanium cylinder coated with the semiconductive material as the working electrode. Illumination of the cell was achieved using a halogen lamp emitting characteristic white light with an intensity of 1000 W/m^2^, directed through a quartz window.

## 3. Results and Discussion

### 3.1. XRD Investigation of the Thin Films

**Figure 2:**
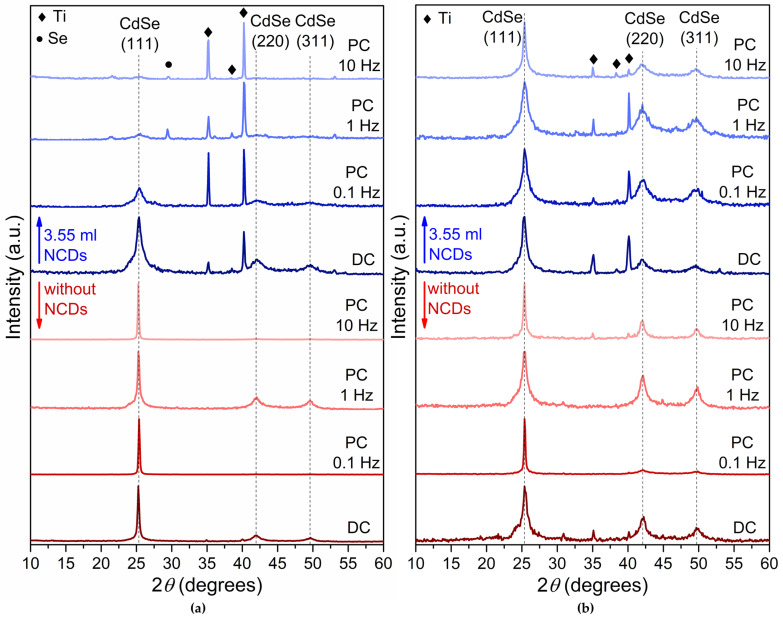
XRD results for thin films deposited from an acidic aqueous solution under −0.6 V/SCE (**a**) and −0.7 V/SCE (**b**). Red colored diffractograms are without addition of NCDs, while blue colored diffractograms are with the addition of 3.55 mL NCDs. The specimens exhibit cubic zinc-blende structure indicated by an intensity peak at about 2*θ* = 25° for the (111) planes. In some diffractograms, the titanium substrate is also visible by the peaks found at 2*θ* 35° and 40.4°.

Figure 2 depicts the X-ray diffraction spectra of plain CdSe thin films (in red color) and CdSe thin films produced with the addition of the NCDs solution in the electrolytic bath (in blue color) deposited under −0.6 V/SCE (Figure 2a) and −0.7 V/SCE (Figure 2b) using both DC and PC.

The plain CdSe thin films exhibit a crystalline structure corresponding to the metastable cubic zinc-blende phase, in agreement with previous reports [1,2,3], as confirmed by the characteristic (111), (220), and (311) reflections of cubic CdSe (ICDD PDF 65-2891). Moreover, in most of the plain CdSe thin film XRD spectra, the (220) and (311) diffraction peaks are also present. Diffraction peaks associated with the titanium substrate (ICDD PDF 44-1294) are also present, particularly in thinner samples. Notably, the substrate peaks become more pronounced in the hybrid films, which is consistent with their relatively reduced thickness (Table 1) and the lower deposition current caused by NCDs incorporation, as described in Section 2.2. Reflections attributed to elemental selenium (ICDD PDF 06-0362) are detected in some specimens, particularly those deposited at higher PC frequencies, indicating that Se segregation can occur under specific electrodeposition conditions.

The hybrid thin films developed by the electrolytic bath containing 3.55 mL of the NCDs solution exhibit a cubic zinc-blende structure similar to that observed for their plain CdSe counterparts. However, the NCDs seem to modify the distribution of the crystallites along the (111), (220) and (311) planes by limiting the presence of the two last ones. The (220) and (311) planes are notable at the hybrid thin films obtained under DC and low pulse frequency PC conditions, namely at frequencies of 0.1 Hz and 1 Hz, especially under −0.6 V. It is believed that the incorporation of NCDs disrupts the development of (111) texture by increasing the density of heterogeneous nucleation sites and altering local interfacial electric fields. These effects promote isotropic nucleation, limit crystallite coalescence, and suppress the thermodynamic preference for (111) plane development. Consequently, the hybrid films show a weaker (111) preferential orientation, regardless of the pulse frequency.

Moreover, the observed peak broadening indicates that the incorporation of NCDs into the electrolytic solution results in the production of finer grain-to-amorphous thin films. Particularly noteworthy is the broadening and decreased intensities of the (111) diffraction peaks in the hybrid XRD spectra (Figure 2, blue color diffractograms) compared to their plain CdSe counterparts (Figure 2 red color diffractograms), suggesting the development of finer-grained semiconductive thin films [1,26]. The modification in crystalline development suggests that the introduction of the NCDs influences the growth dynamics of the CdSe lattice and alters the electro-crystallization process of the CdSe thin films. To quantify these effects, the crystallite size (*D_hk_*_l_) was determined using the Debye–Scherrer equation:

(2) $$D_{hkl}=\frac{K\cdot \lambda }{FWHM\cdot cos\theta }$$

where *λ* = 1.54 A˚ the Cu*K*_α_ wavelength, *θ* the Bragg angle, and *Κ* Scherrer’s constant (commonly 0.94 for spherical crystals with cubic symmetry). Gaussian fitting was used to extract accurate *FWHM* values. The results are summarized in Table 2.

At −0.6 V/SCE, the plain CdSe films exhibit crystallite sizes of 25–45 nm, whereas the hybrid films show consistently smaller values (approximately 6.8–11.5 nm), confirming the pronounced grain-refining effect of NCDs. This contrast becomes even clearer under pulsed deposition, where the plain films retain comparatively large crystallites, while the hybrid films maintain substantially reduced dimensions. At −0.7 V/SCE, the higher cathodic overpotential leads to faster nucleation, resulting in generally smaller crystallites for both systems compared with −0.6 V/SCE. The hybrid films remain smaller overall (8.7–16.8 nm), and their crystallite size increases with increased frequency. This behavior suggests that higher pulse frequencies promote short, repeated nucleation–growth cycles that allow limited merging of nanocrystallites, whereas lower frequencies favor the formation of finer structures. The plain CdSe films display crystallite sizes of 11.5–27.8 nm, with the smallest values occurring at 1 Hz. It seems that in the absence of NCDs at low frequency (0.1 Hz), the long off-time allows for substantial ion replenishment and surface relaxation, which favors the growth of larger crystallites as adatoms have sufficient mobility to reorganize into energetically preferred orientations. At 1 Hz, the shorter but still significant off-times increase the density of nucleation events while limiting crystallite coalescence, resulting in the smallest crystallite sizes measured. At 10 Hz, the system experiences rapid on/off cycles that restrict complete relaxation and promote a higher population of partially grown nuclei, some of which merge during successive pulses, leading again to an increase in crystallite size. Overall, the Scherrer analysis demonstrates that NCDs incorporation systematically suppresses crystallite growth under all conditions, while the combined effects of overpotential and pulse frequency govern the balance between nucleation and the growth of larger crystallites.

Finally, the appearance of elemental Se in some hybrid films can be rationalized by the underlying electrodeposition mechanisms. CdSe formation in selenite electrolytes involves three coupled reactions:

(3) $$Cd+4H^{+}+H_{2}SeO_{3}+4e^{-}↔CdSe+3H_{2}O$$

(4) $$Se+2H^{+}+2e^{-}↔H_{2}Se$$

(5) $$2H_{2}Se+H_{2}SeO_{3}↔3Se+3H_{2}O$$

While Reaction (3) forms CdSe, Reactions (4) and (5) generate elemental selenium. The selective formation of elemental Se predominantly in hybrid films can be rationalized by the role of NCDs in locally altering the electrochemical environment. NCDs possess surface nitrogen functionalities that act as electron-rich centers, facilitating localized charge accumulation during the cathodic pulse. This shifts the relative overpotentials of the competing reduction pathways, making Reactions (4) and (5) kinetically favored in regions where NCDs are embedded. Under pulsed-current conditions, especially at higher frequencies, the rapid alternation between nucleation bursts and diffusion-limited relaxation enhances this effect, enabling discrete Se-rich nanodomains to form. This mechanistic interpretation aligns with both the intensified Se peaks in XRD and the higher Se at% detected by EDS for hybrid films. The excessive selenium content can be removed through appropriate thermal treatment [1,3].

### 3.2. SEM and SEM-EDS Investigation of Thin Films

**Figure 3:**
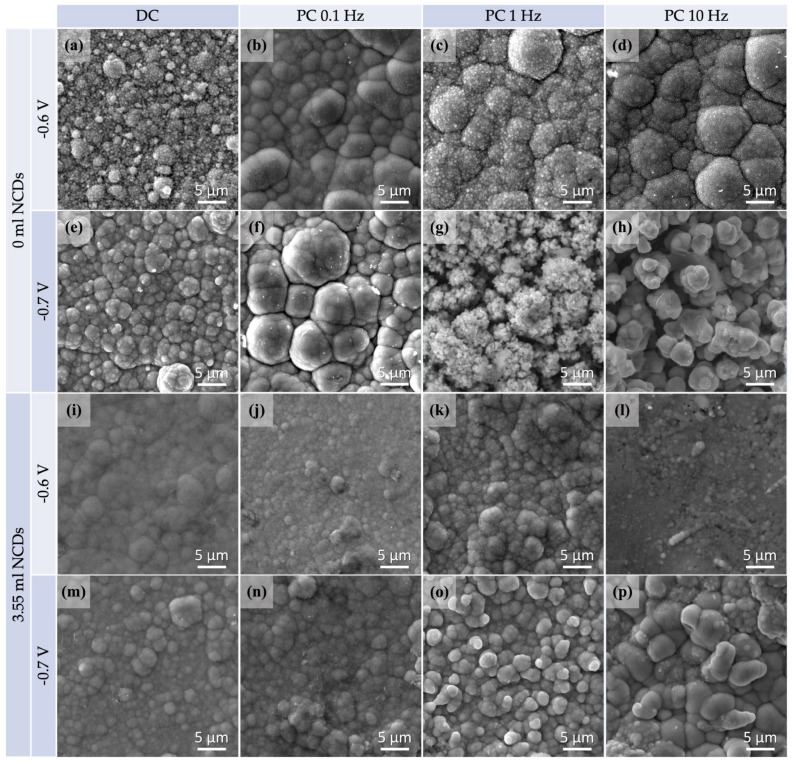
SEM micrographs for specimens plated from an acidic aqueous solution at −0.6 V/SCE and −0.7/SCE both at DC and PC regimes, with no addition of NCDs (**a**–**h**) and with the addition of 3.55 mL NCDs (**i**–**p**).

All the plain and hybrid CdSe thin films were examined by SEM imaging and EDS analysis. The SEM micrographs are depicted in Figure 3 and the results of the EDS analysis are presented in Table 3. In the Supplementary Materials, indicative EDS spectra for each thin film can be found.

Table 3 includes the carbon content detected by EDS for specimens fabricated both in the absence and in the presence of NCDs in the electrolytic bath. Since all compounds introduced into the bath are non-carbonaceous, the carbon detected in the NCD-free thin films is attributed to sources unrelated to the coating itself, such as hydrocarbon accumulation during SEM examination or contamination occurring in the interval between fabrication and examination. The nearly twofold increase in carbon content observed at the specimens fabricated at the bath containing 3.55 mL of NCDs is therefore attributed to the successful incorporation of the NCDs into the coating.

Moreover, the incorporation of NCDs within the hybrid material was further confirmed by Fourier Transform Infrared (FT-IR) spectroscopy, as indicated by the characteristic vibrational bands observed in the corresponding spectrum.

A distinctive cauliflower-like surface morphology typical of the electrodeposited CdSe films can be observed in most of the displayed thin films. In Figure 3a–h, SEM micrographs of plain CdSe thin films are given for comparison purposes. Figure 3i–p depicts SEM micrographs of hybrid thin films deposited with the addition of 3.55 mL NCDs solution. SEM imaging was performed on the as-plated films without applying a gold coating, and the bright spots observed correspond to charging effects typically associated with CdSe surfaces.

Notably, altering the conditions of the electrolytic plating process can induce variations in the structure of the materials. Specifically, the SEM micrographs revealed that by raising the applied voltage or pulse frequency values, more fine grain structures arise. Thin films deposited under DC or low-pulse frequency PC conditions (such as 0.1 Hz) exhibited a more refined grain structure, subsequently resulting in a smoother surface. On the contrary, a composition consisting of small grains was observed in specimens which had been plated at higher voltage and pulse frequency (such as −0.7 V/SCE and 10 Hz) values.

In addition, as indicated by the XRD results, NCDs have contributed to the development of a more complex polycrystalline structure through their inclusion in the CdSe matrix. The resulting hybrid thin films exhibit a finer grain structure that deviates slightly from the conventional cauliflower morphology, displaying a smoother appearance with less refined grain boundaries. It is worth mentioning that the plain CdSe thin film prepared under conditions of −0.7 V/SCE and 10 Hz exhibits dendritic morphological features, likely attributed to an excess of Cd, as confirmed by the EDS results of Table 2 [26]. A similar morphology is observed for the hybrid thin film plated under identical conditions, featuring oval-shaped large crystallites, despite the low Cd at% according to EDS analysis.

The EDS analysis of hybrid thin films confirms the presence of carbon, indicating the successful incorporation of NCDs into the CdSe matrix. A large shift in Cd and Se balance has occurred for hybrid thin films, which results in a marked enrichment of the Se element. Thin films plated from the electrolytic solution with the addition of NCDs exhibit higher concentrations of Se, as shown by their EDS spectra, in comparison to the plain CdSe deposits. Consequently, the presence of the NCDs in the electrolytic bath triggers the nucleation and subsequent growth of nano-crystallites composed solely of Se, as supported also by the XRD findings.

Although the same amount of NCDs was added to all electrolytes, the detected carbon at% varies across deposition conditions. This variability arises from differences in NCD uptake efficiency, which depend strongly on the applied potential and pulse frequency. At more cathodic potentials and higher frequencies, the rapid nucleation bursts favor the entrapment of carbonaceous species within growing CdSe clusters, whereas under milder or more diffusion-controlled conditions the NCDs remain more dispersed in the electrolyte, resulting in lower incorporation. Additionally, the competitive nucleation between CdSe and Se-rich phases alters the number of available binding sites for NCD attachment. These effects collectively explain the non-uniform carbon incorporation despite the identical solution concentration.

To further examine the influence of NCDs on film thickness, cross-sections were prepared for two characteristic samples fabricated with and without the addition of NCDs in the electrolytic bath, under identical electrodeposition parameters, namely in the DC regime at −0.6 V/SCE and for the same deposition time.

**Figure 4:**
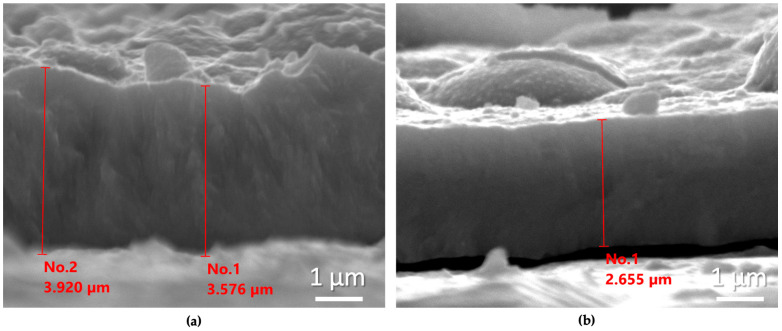
SEM micrographs of the cross-sections of the coatings prepared at the DC regime at –0.6 V/SCE without (**a**) and with (**b**) the addition of NCDs at the electrolytic bath.

In Figure 4, representative SEM micrographs of the cross-sections of these two samples are shown.

It is clearly observed that the coating fabricated at the electrolytic bath containing NCDs exhibits a reduced thickness of approximately 1 μm. Furthermore, it is important to emphasize the very good agreement between the estimated thickness using the 2nd Faraday law, as presented in Table 1, and those obtained experimentally.

### 3.3. Investigation of Thin Films Band Gap

**Figure 5:**
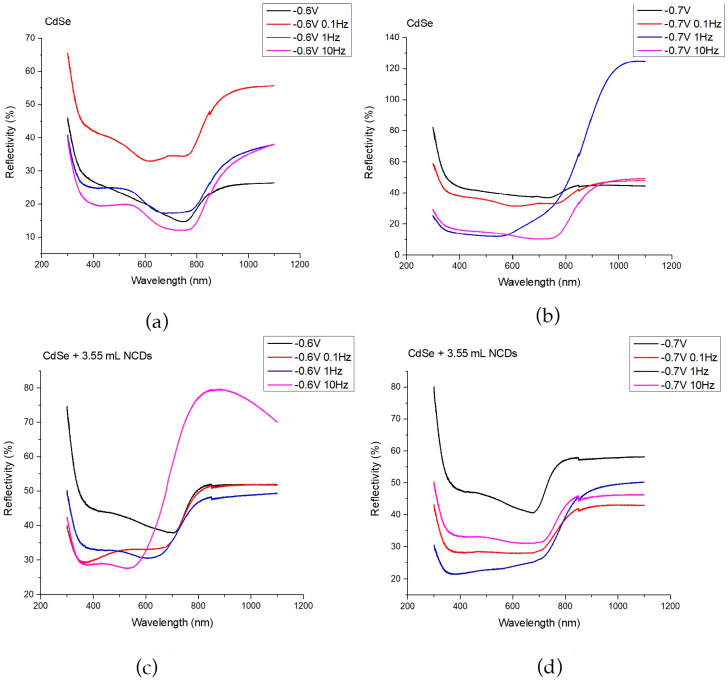
UV-Vis spectra for thin films deposited from an acidic aqueous solution under −0.6 V/SCE (**a**) and −0.7 V/SCE (**b**), the same aqueous solution with the addition of 3.55 mL of NCDs solution under −0.6 V/SCE (**c**) and −0.7 V/SCE (**d**). The specimens were developed in DC and PC regimes.

The energy gap of each thin film was determined using a UV-Vis spectrometer equipped with an integration sphere, allowing the collection of reflectance spectra from each specimen. The corresponding spectra are presented in Figure 5, showing the UV–Vis response of thin films deposited from an acidic aqueous solution under –0.6 V/SCE (a) and –0.7 V/SCE (b), as well as from the same solution containing 3.55 mL of NCDs solution under –0.6 V/SCE (c) and –0.7 V/SCE (d), for specimens prepared under both DC and PC regimes.

The band gaps were calculated from the absorption edge using the Kubelka–Munk (K–M) approach and the corresponding Tauc plots [2,7]. Since the measurements were performed in diffuse reflectance mode on non-transparent, irregular electrodeposited films where transmission is negligible, reflectance was the only reliable optical signal. For this reason, the K–M method was followed rather than, for example, the Beer–Lambert law, which requires a uniform, transmitting film with a defined optical path length. Therefore, K–M provided the most appropriate estimate of the absorption coefficient for band-gap determination under the present measurement geometry. The calculated results are presented in Table 4.

In Figure 5a,b, the reflectivity spectra of plain CdSe thin films are presented for the prepared coatings at –0.6 and –0.7 applied voltages, respectively. A decrease in reflectivity and a simultaneous increase in absorption within the wavelength range of approximately 400 nm to 800 nm is revealed, with energy gaps ranging from 1.58 eV to 1.73 eV. Since these variations do not exceed the measurement uncertainty (±0.05 eV), the differences between the plain CdSe samples under all voltages and pulse frequencies are not considered significant, indicating that the electronic structure of CdSe remains essentially unaffected in the absence of NCDs.

In the case of hybrid thin films deposited at –0.6 V/SCE, a blue shift towards shorter wavelengths at the absorption edge was observed, resulting in an accompanying increase in the energy gap. Reflectance spectra in Figure 5c demonstrates an increased absorbance within the range of 350 nm to 700 nm, while the energy gaps range from 1.72 eV to 2.10 eV. This observation can be attributed to the increased absorption at shorter wavelengths (λ < 350 nm) exhibited by NCDs [13,22,28]. Specifically, the band gap increases systematically with pulse frequency, exceeding the measurement variability, which suggests that the moderate deposition rate under this potential allows more effective incorporation and interaction of NCDs within the CdSe matrix. Higher pulse frequencies are expected to promote finer structural features, enhanced confinement, defect redistribution, and charge-transfer effects, all contributing to a shift in the absorption edge toward shorter wavelengths and the widening of the band gap [14,18,32,33].

For the hybrid films deposited at –0.7 V/SCE, the band gap is slightly higher than that of the corresponding plain CdSe films, but it does not exhibit any significant dependence on pulse frequency, remaining within 1.72 to 1.80 eV, a range that lies inside the experimental uncertainty (±0.05 eV). The modest increase relative to plain CdSe can be attributed to the incorporation of NCDs, which introduce additional defect-related states and subtle structural modifications that can widen the optical gap to a limited extent. However, at this more cathodic deposition potential, the nucleation and growth of CdSe proceed more rapidly and with reduced diffusion control, limiting the influence of the pulse–rest sequence on the evolving electronic transitions. As a result, variations in pulse frequency do not produce measurable shifts in the absorption edge. Overall, band gap tuning through pulse frequency in the presence of NCDs becomes evident only at –0.6 V/SCE, where the lower overpotential allows the deposition dynamics and the resulting optical response to remain sensitive to the applied waveform.

### 3.4. Photoelectrochemical Behavior

**Figure 6:**
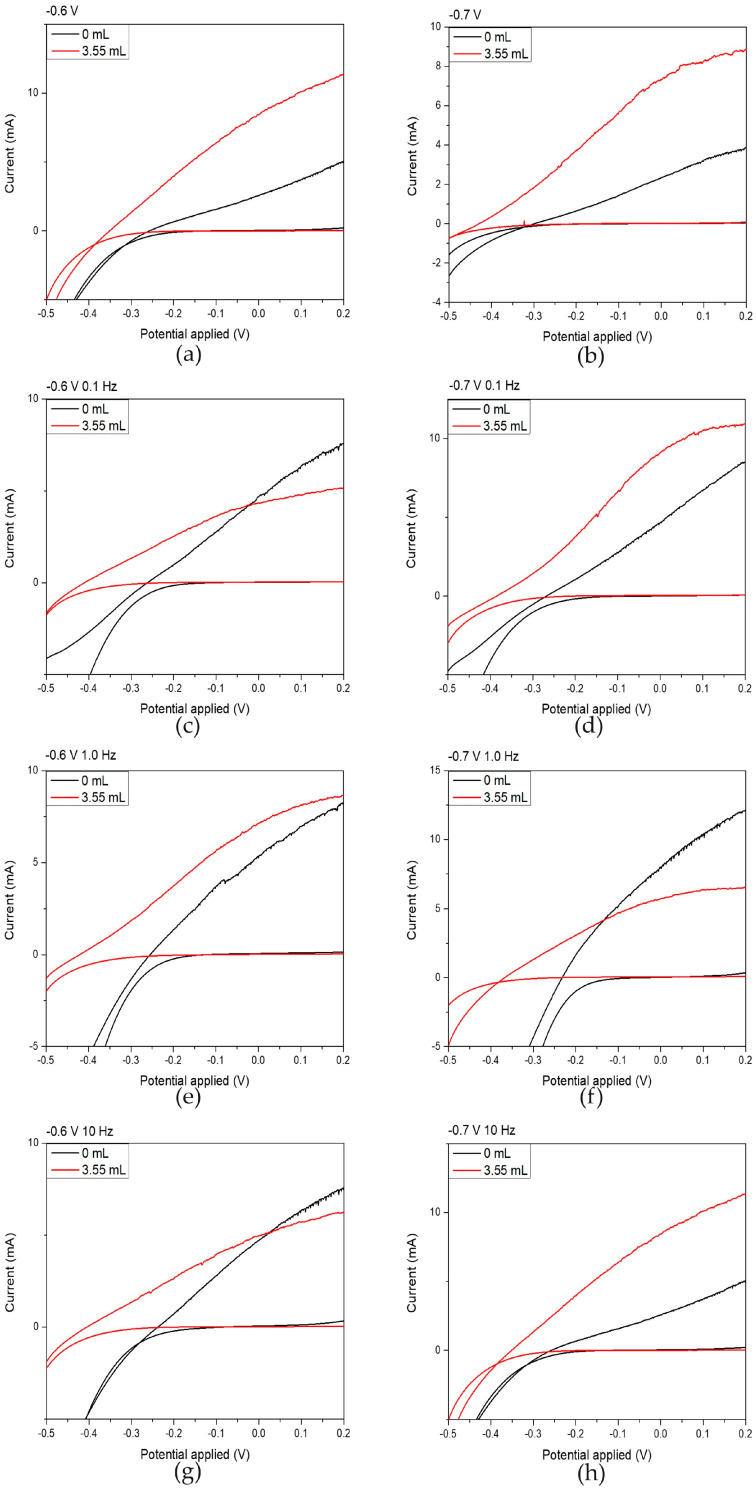
Electrochemical photoelectrochemical (PEC) potential and photoinduced current responses under illumination of 1000 W/m^2^ for thin films prepared from an acidic aqueous solution by varying the electrodeposition parameters. Black curves correspond to films prepared without NCDs, while red curves correspond to films prepared with the addition of 3.55 mL NCDs to the deposition bath. Graphs (**a**,**c**,**e**,**g**) show measurements recorded at an applied potential of −0.6 V/SCE with modulation frequencies of 0.1 Hz, 0.1 Hz, 1.0 Hz, and 10 Hz, respectively, whereas graphs (**b**,**d**,**f**,**h**) show measurements recorded at −0.7 V/SCE with modulation frequencies of 0.1 Hz, 0.1 Hz, 1.0 Hz, and 10 Hz, respectively.

The photoelectrochemical response of each specimen was investigated using a conventional three-electrode configuration, as illustrated in Figure 1. The current-voltage (I–V) characteristics of the CdSe thin-film electrodes under illuminated and dark conditions are presented in Figure 6. The short-circuit current ($I_{SC}$) and open-circuit voltage ($V_{OC}$) were obtained directly from the I–V curves, while the fill factor (FF), and photoelectrochemical efficiency (η%) were determined using Equations (6) and (7):

(6) $$FF=\frac{P_{max}}{I_{SC}\times V_{OC}}$$

(7) $$\eta \%=\frac{I_{SC}\times V_{OC}}{P_{in}\times A}\times FF\times 100\%$$

where $P_{max}$ is the maximum power of the thin film electrode,${\;P}_{in}$ is the incident light power and A is the illuminated active area of the photoelectrode. All current values were normalized to the active illuminated area (A = 1.13 cm^2^) to obtain the current density (*J*). The corresponding photoconversion parameters, including short-circuit current density ($J_{SC}$), open-circuit voltage ($V_{OC}$), fill factor (FF), and photoelectrochemical efficiency (*η*%), are summarized in Table 5. All recorded photocurrents exhibit anodic behavior, confirming the *n*-type semiconducting nature of the CdSe electrodes [1,8,12].

Upon careful examination of the results included in Table 5, it becomes evident that hybrid thin films exhibited a marked improvement in their photoelectrochemical behavior compared to the plain CdSe thin films. This enhancement is particularly pronounced in thin films plated under DC conditions at −0.6 V/SCE and −0.7 V/SCE, where the photoelectrochemical efficiency increased by factors of 3.5 and 3.7, respectively, relative to their CdSe counterparts. A significant increase in efficiency is also observed for films deposited under pulsed-current conditions at −0.7 V/SCE, especially at 0.1 Hz and 10 Hz.

The origin of these improvements can be attributed to several interconnected effects introduced by the successful incorporation of NCDs, as evident from the EDS analysis. Their presence promotes more efficient charge separation by reducing electron–hole recombination and by providing additional conductive pathways for electron transport, which accounts for higher JSC values. The hybrid films also develop a finer-grained microstructure and cauliflower-like surface morphology, as evidenced by XRD and SEM analysis. Such structural refinement increases the interfacial area and shortens carrier diffusion distances, enhancing charge-transfer kinetics at the semiconductor–electrolyte interface and contributing to the improved fill factor. A mild Se excess is also observed in hybrid films, which reduces Cd-vacancy defects and enhances n-type conductivity, further improving charge separation and supporting the superior PEC performance. Under pulsed deposition at −0.7 V/SCE, the periodic modulation of the potential influences the incorporation and distribution of NCDs within the CdSe matrix, promoting more favorable interfacial electronic interactions and explaining the enhanced performance at 0.1 Hz and 10 Hz.

It is also important to note that in electrochemically deposited CdSe the minority carrier diffusion length is significantly shorter than the total film thickness, typically extending only a few tens to a few hundred nanometers from the illuminated surface. As a consequence, photocurrent generation is governed primarily by this near-surface region, whereas carriers generated deeper within the film undergo rapid recombination and do not contribute effectively to the PEC response. Thickness variations within the range of 1–4 μm are therefore not expected to dictate the observed improvements in performance. The superior behavior of the hybrid films arises instead from improved charge transport, reduced recombination, and the favorable microstructure induced by NCD incorporation.

More specifically, the thin films plated from the electrolytic bath containing 3.55 mL of the NCDs solution and exhibiting the best photovoltaic performance were those produced under two conditions: (a) At −0.6 V/SCE in the DC regime, the hybrid film displayed an EDS composition of Cd = 23.1 ± 1.3 at%, Se = 34.2 ± 1.9 at%, and C = 42.7 ± 3.2 at%, and achieved a 3.5-fold increase in *η* compared with the corresponding plain CdSe film. (b) When plated under PC −0.7 V/SCE 10 Hz, the hybrid thin film demonstrated a composition comprising Cd = 21.8 ± 1.1 at%, Se = 42.3 ± 1.0 at%, and C = 35.9 ± 1.9 at%, resulting in a 3.8 times improvement in photovoltaic efficiency. Despite the substantial C contribution, the Cd and Se fractions maintain a near-stoichiometric balance with a mild Se excess, a condition known to suppress Cd-vacancy defects, enhance *n*-type conductivity, and promote more efficient charge separation—consistent with the superior PEC response measured for these hybrid films.

These enhanced efficiencies are consistent with our previous findings, where similar improvements were observed when using the electrodeposition process for the development of hybrid multilayer semiconductors in the presence of organic additives associated with the formation of hexagonal CdSe [12]. Notably, in the present work, the photoelectrochemical behavior is further improved under the aforementioned conditions, even though the films retain the cubic zinc-blende structure, indicating that NCDs incorporation beneficially modifies the microstructure and electronic properties of the film, independently of any transformation to the hexagonal phase.

## 4. Conclusions

This is an initial effort to develop novel hybrid semiconductors by incorporating NCDs into a CdSe matrix. The NCDs were synthesized using the hydrothermal method. Hybrid semiconducting thin films were prepared through a one-step electrocodeposition technique. The full characterization of the specimens revealed a cubic zinc-blende structure and cauliflower texture, similar to CdSe.

The addition of NCDs to the CdSe matrix resulted in several differentiations in the materials’ physicochemical properties. There was a finer grain structure, an increase in the selenium concentration, and an increase in the energy gap of the semiconductor. The influence of the additive on the thin films varied depending on the amount of incorporation of NCDs and their distribution into the lattice, as well as the electrochemical conditions applied during the plating process. Notably, the majority of the specimens exhibited significantly improved photoelectrochemical properties compared to their plain CdSe counterparts. This behavior could be attributed to the fact that the NCDs can create crystal defects on the CdSe deposits, acting as electron acceptors, and resulting in a positive impact to the photoconductivity of the specimens.

In conclusion, the incorporation of NCDs has proven to be a promising avenue for enhancing the overall efficiency of the photoelectrochemical cell. The observed trends, especially characterizing the best-performing specimens, and the influence of plating conditions, have shed light on potential mechanisms governing their enhanced performance. By understanding the correlation between lattice structure and material properties, we aim to optimize the performance and durability of these hybrid semiconductors, propelling advancements in sustainable energy technologies, and environmental remediation. This research marks a significant step forward in the pursuit of efficient photoelectrochemical devices, and further exploration into the underlying principles will undoubtedly stimulate the development of advanced and sustainable materials for future energy applications.

## Figures and Tables

**Table 1:** Electrodeposition parameters.

Applied Voltage (V/SCE)	Pulse Frequency (Hz)	Duty Cycle (%)	NCDs Addition (mL)	Film Thickness * (μm)
−0.6	DC	-	0	3.85
	0.1	70	0	3.53
	1.0	70	0	3.74
	10	70	0	3.58
	DC	-	3.55	2.92
	0.1	70	3.55	2.03
	1.0	70	3.55	1.81
	10	70	3.55	1.60
−0.7	DC	-	0	3.02
	0.1	70	0	3.64
	1.0	70	0	2.74
	10	70	0	2.98
	DC	-	3.55	2.68
	0.1	70	3.55	2.12
	1.0	70	3.55	2.81
	10	70	3.55	2.40

* Thickness values estimated using Faraday’s law; uncertainty approximately ±0.3 μm.

**Table 2:** Scherrer crystallite sizes, FWHM values, and (111) peak positions for CdSe thin films deposited at −0.6 and −0.7 V/SCE under DC and PC conditions, with and without NCDs.

		2*θ* Peak (°)		*FWHM* (°)		*D_hkl_* (nm)	
NCDs (mL)		0	3.55	0	3.55	0	3.55
0.6 V/SCE	DC	25.3	25.5	0.298	0.800	29.8	11.5
	0.1 Hz	25.4	25.6	0.215	0.954	39.5	6.8
	1.0 Hz	25.3	25.9	0.345	1.584	25.2	7.3
	10 Hz	25.3	25.0	0.197	1.527	45.0	10.1
0.7 V/SCE	DC	25.8	25.5	0.686	0.736	11.9	11.1
	0.1 Hz	25.4	25.7	0.240	1.080	20.7	8.9
	1.0 Hz	25.4	25.7	0.765	1.169	11.5	8.7
	10 Hz	25.3	25.4	0.379	0.843	27.8	16.8

**Table 3:** EDS results (in at%).

Elements		Cd		Se		C	
NCDs (mL)		0	3.55	0	3.55	0	3.55
0.6 V/SCE	DC	34.5 ± 1.3	23.1 ± 1.3	44.3 ± 1.9	34.2 ± 1.9	21.2 ± 3.1	42.7 ± 3.2
	0.1 Hz	37.4 ± 2.2	23.4 ± 1.4	41.7 ± 1.8	38.4 ± 0.8	20.9 ± 3.9	38.2 ± 2.1
	1.0 Hz	37.6 ± 0.4	20.2 ± 0.9	42.5 ± 0.7	36.0 ± 3.2	19.9 ± 1.1	43.8 ± 3.8
	10 Hz	39.4 ± 1.3	11.4 ± 1.2	42.2 ± 1.6	26.7 ± 3.2	18.4 ± 2.2	61.9 ± 4.3
0.7 V/SCE	DC	41.9 ± 2.6	35.6 ± 1.5	41.3 ± 1.0	34.9 ± 0.9	16.8 ± 1.7	29.5 ± 1.3
	0.1 Hz	37.2 ± 2.3	19.9 ± 3.1	41.1 ± 1.6	36.9 ± 5.3	21.7 ± 3.8	43.2 ± 8.3
	1.0 Hz	25.9 ± 2.9	20.9 ± 0.8	53.7 ± 4.1	41.5 ± 1.8	20.4 ± 6.5	37.6 ± 2.5
	10 Hz	52.6 ± 9.5	21.8 ± 1.1	28.1 ± 6.1	42.3 ± 1.0	19.3 ± 6.0	35.9 ± 1.9

**Table 4:** Energy band gap of specimens deposited from the electrolytic bath with no addition of the NCDs solution as well as with the addition of 3.55 mL of the aforementioned solution. The specimens were plated under DC and PC conditions.

Voltage (V/SCE)	Pulse Frequency (Hz)	Band Gap (eV)	
		Plain CdSe	CdSe + 3.55 mL NCDs
−0.6	DC	1.63	1.74
	0.1	1.61	1.80
	1.0	1.59	1.88
	10	1.58	2.10
−0.7	DC	1.65	1.80
	0.1	1.62	1.76
	1	1.73	1.72
	10	1.63	1.73

**Table 5:** Photoelectrochemical parameters of CdSe electrodeposits prepared under DC and PC regime from the electrolytic bath with no addition of the NCDs solution as well as with the addition of 3.55 mL of the aforementioned solution.

Photoelectrochemical Response									
		*η* %		*FF*		(mA/cm^2^)		(V)	
NCDs (mL)		0	3.55	0	3.55	0	3.55	0	3.55
−0.6 V/SCE	DC	0.39	1.77	0.29	0.21	3.85	17.56	−0.33	−0.46
	0.1 Hz	0.28	0.47	0.24	0.29	4.15	3.81	−0.26	−0.41
	1 Hz	0.37	0.71	0.29	0.25	4.74	6.31	−0.25	−0.42
	10 Hz	0.27	0.51	0.26	0.27	4.17	4.40	−0.24	−0.40
−0.7 V/SCE	DC	0.15	0.71	0.22	0.24	2.08	6.50	−0.30	−0.43
	0.1 Hz	0.28	0.75	0.24	0.23	4.14	8.09	−0.27	−0.38
	1 Hz	0.53	0.59	0.30	0.30	6.97	5.04	−0.23	−0.37
	10 Hz	0.16	0.77	0.26	0.27	2.26	7.47	−0.26	−0.35

## Data Availability

The original contributions presented in this study are included in the article/Supplementary Materials. Further inquiries can be directed to the corresponding author.

## Supplementary Materials

The following supporting information can be downloaded at: https://www.mdpi.com/article/10.3390/ma18245691/s1, Figure S1: EDS spectra of the coatings without NCDs fabricated at −0.6 V/SCE.; Figure S2: EDS spectra of the coatings without NCDs fabricated at −0.7 V/SCE.; Figure S3: EDS spectra of the coatings with NCDs fabricated at −0.6 V/SCE.; Figure S4: EDS spectra of the coatings with NCDs fabricated at −0.7 V/SCE.; Figure S5: EDS spectra of the coatings with NCDs fabricated at −0.7 V/SCE.
