# Supplementary material for: One-Step Electrodeposition of Hybrid Semiconductive CdSe/Nitrogen-Doped Carbon Dots Thin Films
Source: Materials (Basel). 2025 Dec 18;18(24):5691. doi: 10.3390/ma18245691 (PMC12734489; doi:10.3390/ma18245691)
Supplement: Supplementary file 1 [file materials-18-05691-s001.zip › materials-3997233-supplementary.pdf]

Supplementary Material

One-step Electrodeposition of Hybrid Semiconductive CdSe/ Nitrogen-doped Carbon Dots Thin Films

1. EDS spectra

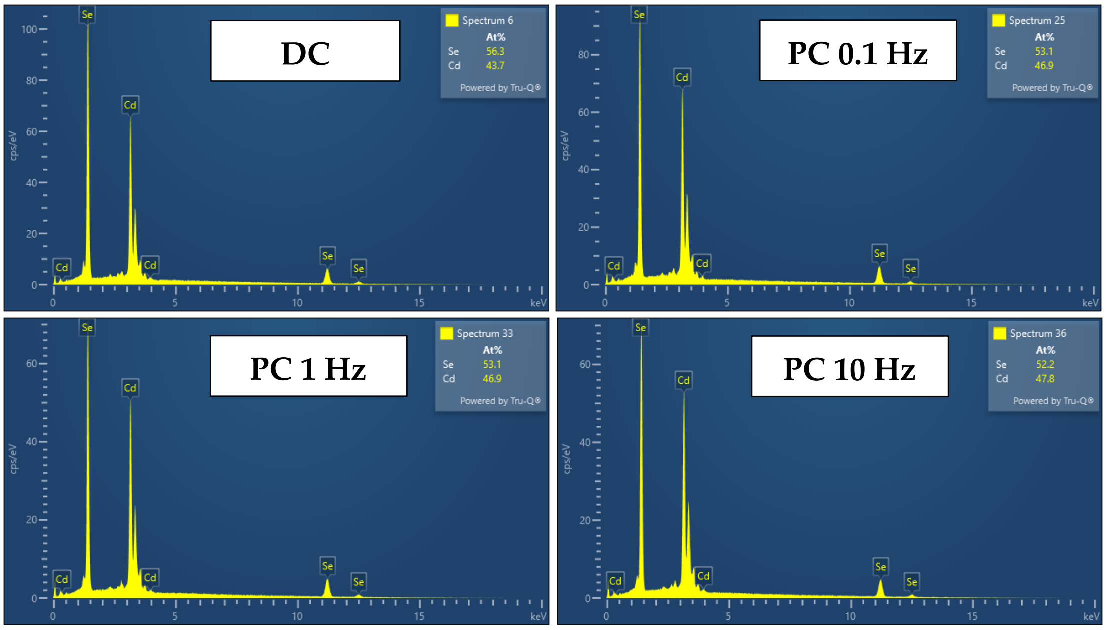

Figure S1. EDS spectra of the coatings without NCDs fabricated at -0.6 V/SCE.

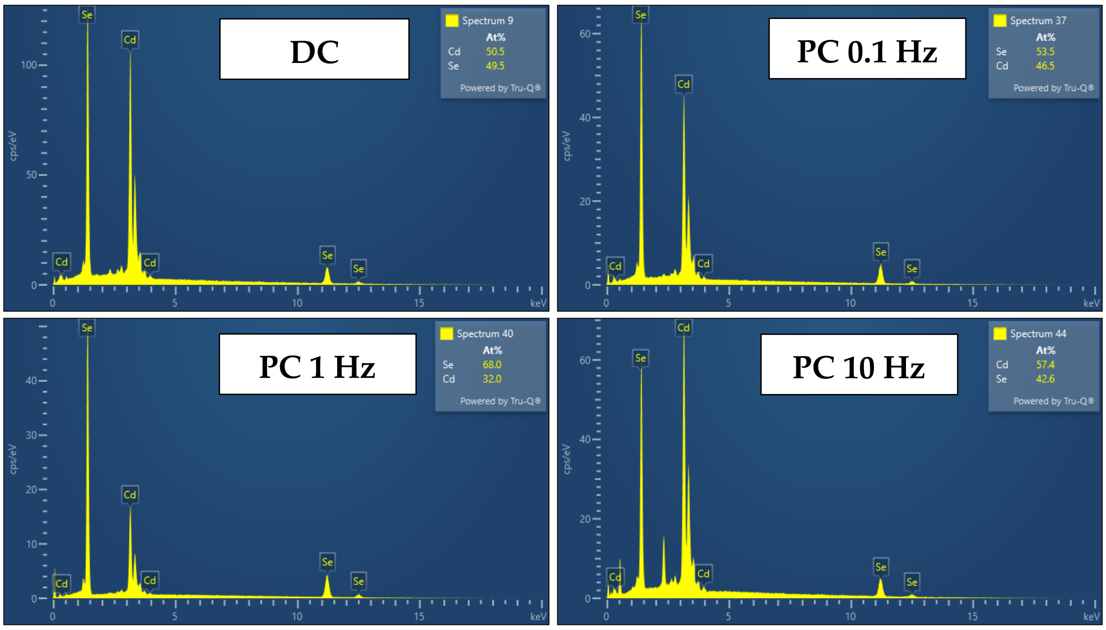

Figure S2. EDS spectra of the coatings without NCDs fabricated at -0.7 V/SCE.

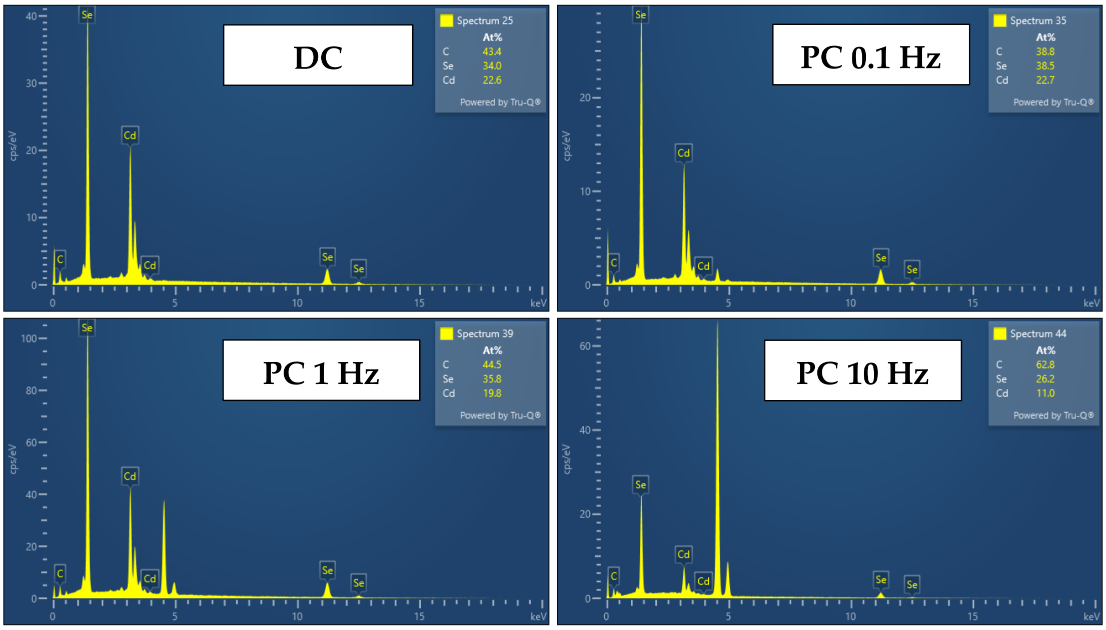

Figure S3. EDS spectra of the coatings with NCDs fabricated at -0.6 V/SCE.

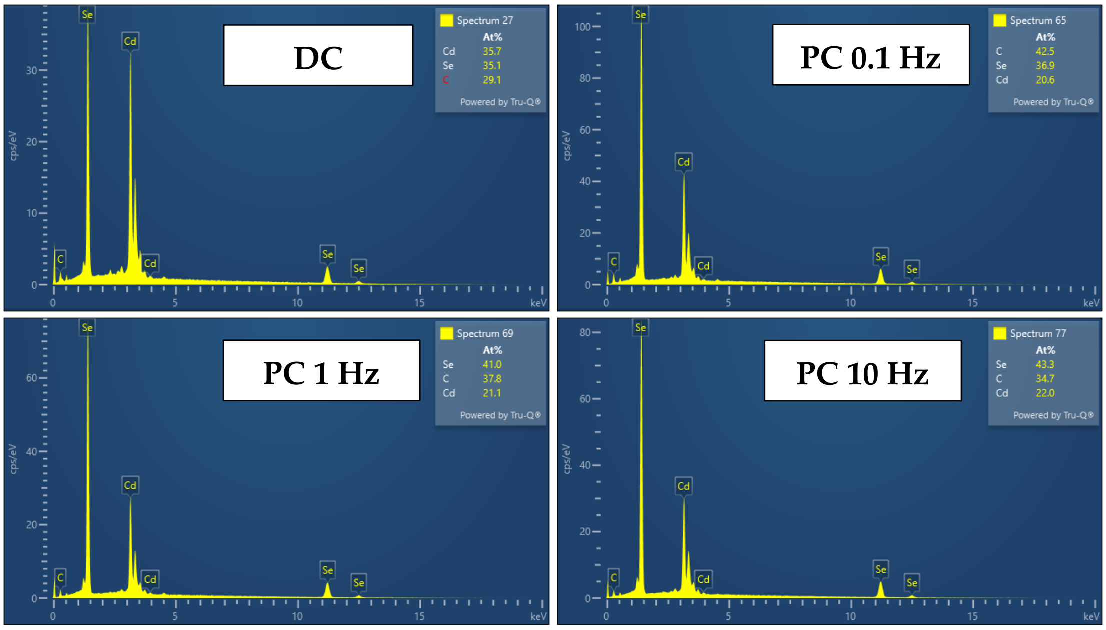

Figure S4. EDS spectra of the coatings with NCDs fabricated at -0.7 V/SCE.

It must be emphasized that the carbon measured at the coatings fabricated at the bath which did not contain NCDs was in the range of 15.4 to 24.7 At%, exhibiting a mean value and a standard deviation of 19.8 ± 2.7 At%.

This presence of carbon, as it is not contained in any substance added in the bath, is attributed to various sources, such as hydrocarbon buildup while examination in the SEM or contamination in the interval between fabrication and examination.

The almost double carbon content measured at the coatings fabricated at the bath with 3.55 mL NCDs is attributed to the embedded NCDs in the coating.

2. FT-IR spectrum

The presence of NCDs was confirmed via Fourier Transform Infrared (FT-IR) spectroscopy. The FT-IR spectrum was obtained with a Jasco FT-IR 4200 spectrometer (Jasco, Japan) in the range of 400-4000 cm^{-1} and resolution of 4 cm^{-1}, using KBr pellets. In order to perform the characterization, the film had to be removed from the substrate and the collected powder was used for pellet formation.

As it is observed in the FT-IR spectrum (Figure S5), the characteristic peak located at 1.631 cm^{-1} is assigned to the C=C stretching vibrations of the carbon core of NCDs. Furthermore, the peaks observed at 2.918 cm^{-1} and 2.850 cm^{-1} are ascribed to the C-H stretching vibrations of CH_2 and CH_3, respectively. In addition, the presence of O-H groups (3.400 cm^{-1}) arises not only from adsorbed water molecules, but also from the abundant functional groups on the surface of NCDs. The peaks located at 1.386 cm^{-1} and 1.093 cm^{-1} correspond to C-OH and C-O-C vibrations, associated with carboxylic and epoxy surface functional groups of NCDs, respectively. Finally, the absorption peaks located at < 1.000 cm^{-1} indicate the presence of CdSe.

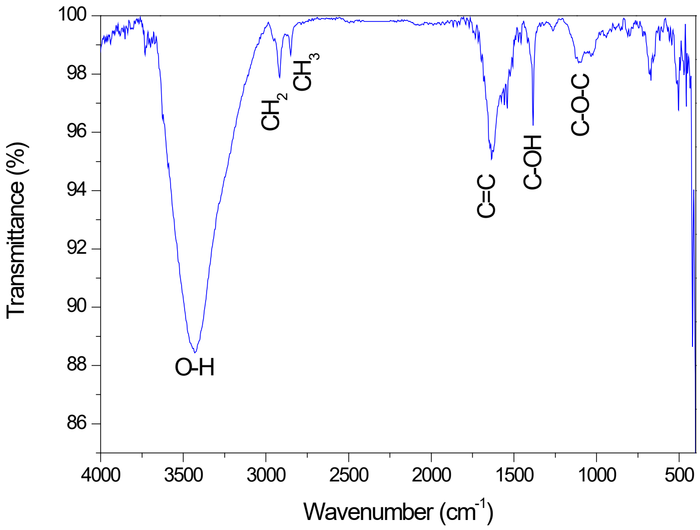

Figure S5. FT-IR spectrum of CdSe/NCDs powder.
